# Ipsilateral femoral neck, intertrochanteric and acetabular fractures with posterior dislocation of the hip: A case report and literature review

**DOI:** 10.1097/MD.0000000000036275

**Published:** 2023-11-24

**Authors:** Dong Liu, Xiaojun Yu, Lin Chen, Zhiqiang Wang

**Affiliations:** a Orthopedic Center 1 Department of Orthopedic Trauma, Suining Central Hospital, Suining City, China.

**Keywords:** acetabulum fracture, femoral neck fracture, intertrochanteric fracture of the femur, posterior dislocation of the hip, proximal femoral locking compression plate (PFLCP)

## Abstract

**Introduction:**

Traumatic hip dislocation with ipsilateral femoral neck fracture, intertrochanteric fracture and acetabular fracture is a rare and serious injury that occurs mostly in young patients and after high-energy trauma. The treatment of these injuries is a major challenge for orthopedic surgeons; it can have devastating consequences if not treated properly, including avascular necrosis of the femoral head and traumatic osteoarthritis of the hip. In previous case reports only internal fixation of femoral neck fracture was performed without revascularisation and there was a lack of long-term follow-up results. In this report, we fixed the fracture revascularised the patient, and obtained good prognostic results at up to 20 months of follow-up.

**Case presentation:**

Here, we report an 18-year-old patient with systemic polytrauma resulting from a car accident. The trauma included ipsilateral posterior hip dislocation, acetabular fracture, femoral intertrochanteric fracture, and femoral neck fracture. In addition, the patient also had an ipsilateral open tibiofibula fracture. We chose the surgical treatment of a proximal femoral locking compression plate (PFLCP), femoral quadratus bone flap graft, and cannulated compression screw to treat the proximal femoral fracture. The patient was followed up for nearly 20 months; the range of motion of his hip was close to normal, and his hip function was good.

**Discussion and Conclusions:**

Traumatic hip dislocation with ipsilateral femoral neck fracture, intertrochanteric fracture and acetabular fracture is a rare and serious injury, and surgical intervention is often needed. Because of the high risk of femoral head necrosis in such complex injuries, it is very important to protect/restore the blood supply of the femoral head during surgery. Therefore, in younger patients, we believe that the use of a quadratus femoris bone flap graft and PFLCP is an acceptable treatment for this severe injury. We discuss the management of this rare case and review the current literature to provide the best evidence-based recommendations for this injury pattern. We conclude that for patients with complex ipsilateral proximal femoral and acetabular fractures combined with hip dislocation, the key to surgical treatment, in addition to anatomic reduction and good fixation, is the primary reconstruction of the femoral head blood supply.

## 1. Introduction

With the rapid development of transportation and industry, the incidence of multiple fractures in adults has become increasingly common, but cases involving ipsilateral acetabulum fracture, femoral neck fracture, and femoral intertrochanteric fracture with posterior dislocation of the hip joint are still rarely reported. The etiology, treatment, prognosis, and patient characteristics of this severe injury are not clearly understood. However, such injuries present challenges for orthopedic surgeons.^[1–3]^ To date, only 2 cases of this type of fracture have been reported.^[4,5]^

Improper treatment of ipsilateral hip dislocation with acetabular fracture, femoral neck fracture, or femoral intertrochanteric fracture can lead to devastating consequences, including avascular necrosis of the femoral head (AVN) and subsequent traumatic osteoarthritis.^[6]^ Browne et al reported 3 cases of this type of injury.^[7]^ Two of them were injured in a traffic accident, and another was injured in a fall from a height. All 3 patients underwent open reduction and internal fixation. However, no prognosis was mentioned. Similar cases were reported by Kuhn et al and Barrett et al^[8,9]^ Both patients were injured in traffic accidents and were treated with open reduction and internal fixation. However, due to the limited sample size, the results may reflect only one or a few aspects of the fracture characteristics. As described by Mediouni et al, the goal of modern orthopedic research is to fill the gap between basic and clinical sciences and to contribute to translational orthopedics.^[10]^

In this article, we report an 18-year-old young patient with concurrent ipsilateral femoral neck fracture, intertrochanteric fracture, posterior acetabular fracture, and posterior hip dislocation. We analyzed the mechanisms of injury and discussed the choice of surgical internal fixation. Based on our clinical experience, we treated this patient with a proximal femoral locking compression plate (PFLCP), quadratus femoris bone flap graft, and cannulated compression screw. After nearly 1 year of follow-up, the patient hip range of motion was close to normal and functioned well.

## 2. Case presentation

**Figure 1.**
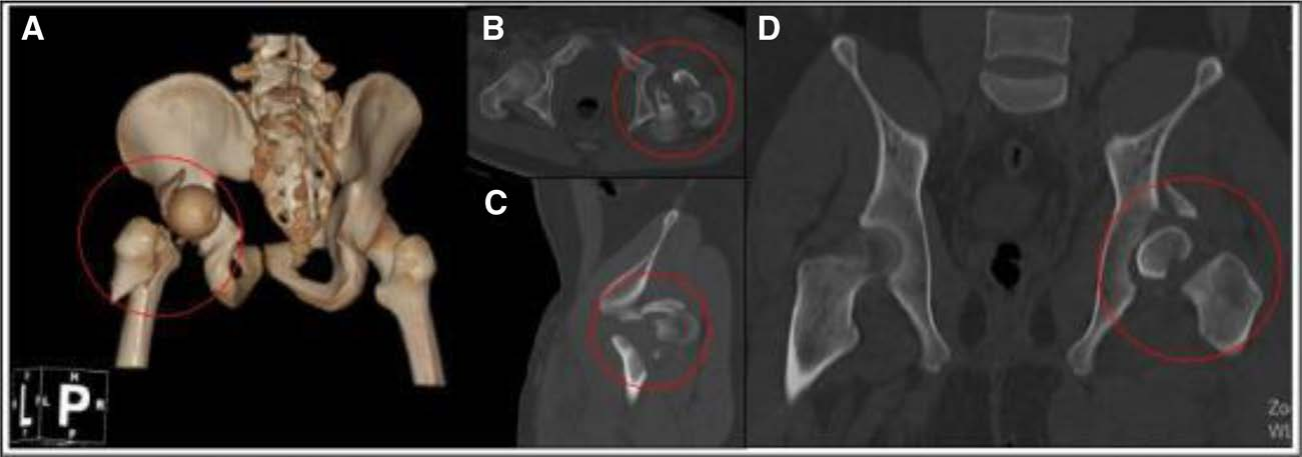
The patient preoperative radiographs and computed tomography images showed ipsilateral femoral intertrochanteric, femoral neck, and acetabular fractures with posterior dislocation of the femoral head. (A) Preoperative pelvic CT 3-dimensional reconstruction; (B) preoperative cross-sectional CT image of the hip joint; (C) preoperative sagittal CT image of the hip joint; (D) preoperative coronal CT image of the hip joint. CT = computed tomography.

**Figure 2.**
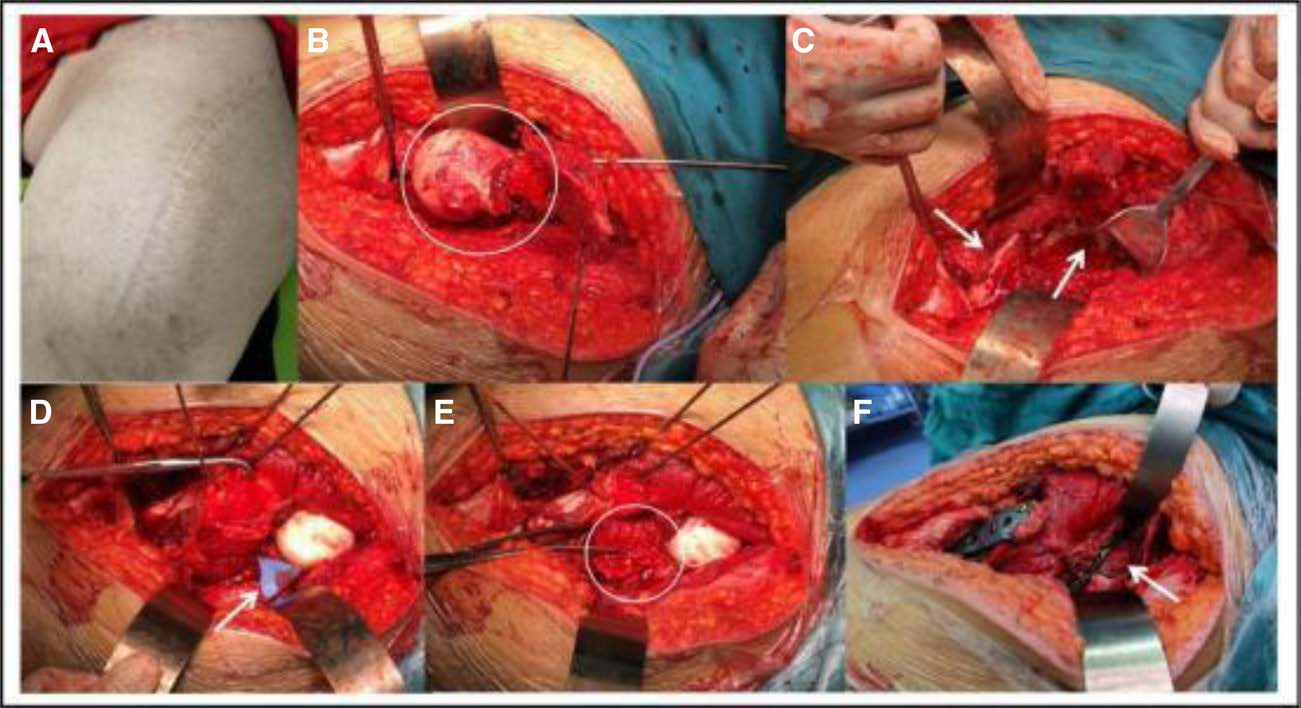
Dislocation and fracture of the femoral head during the operation. (A) Surgical incision and healing; (B, C) femoral neck fracture, dislocation of femoral head and dissociation between muscle fibers; (D, E) the size of the quadratus femoris bone flap was designed according to the fracture condition, and the quadratus femoris bone flap was transplanted into the femoral neck; (F) reduction of the fractured mass and PFLCP/hollow screw fixation of the proximal femur and neck of the femur and reconstruction of the acetabular posterior wall fracture with a plate. PFLCP = proximal femoral locking compression plate.

**Figure 3.**
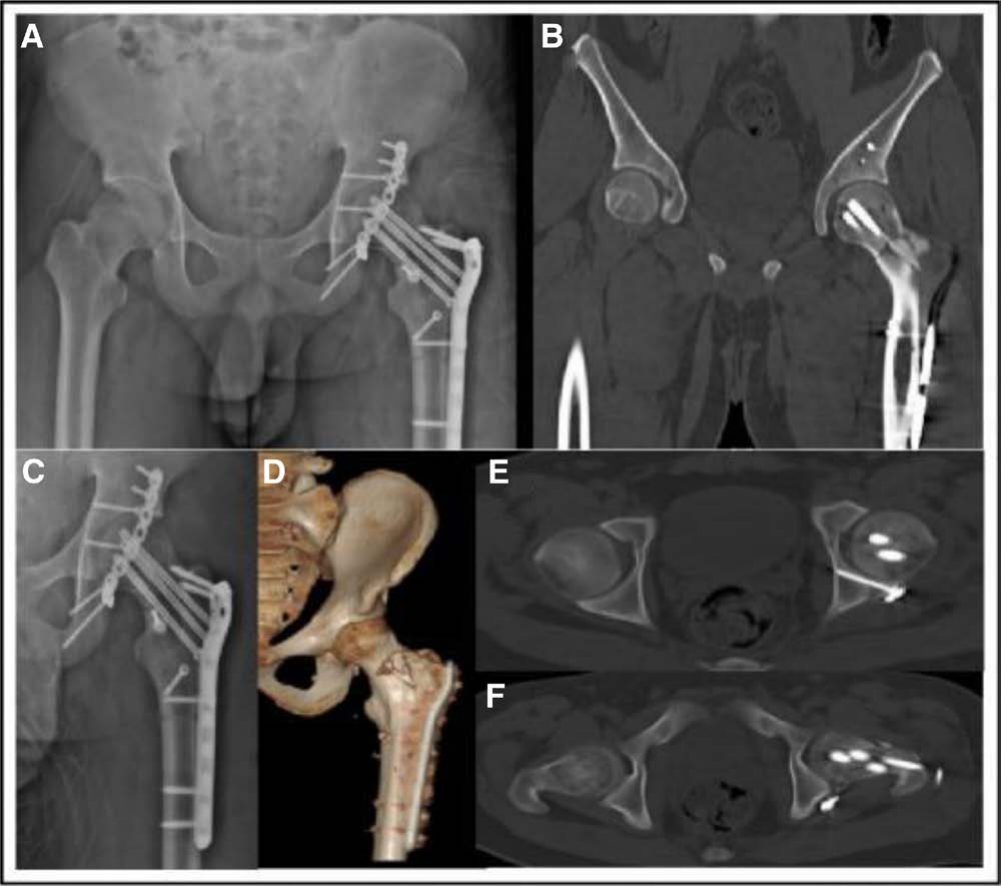
Postoperative DR and CT examination of the pelvis and hip showed that the fracture was well reduced, internal fixation was effective, the screw length was appropriate, and the femoral quadratus bone flap was in a satisfactory position. (B) Postoperative coronal CT images of the pelvis; (C, D) postoperative DR and CT 3D reconstruction of the hip joint; (E, F) postoperative transverse CT images of the pelvis. CT = computed tomography, DR = digital radiography.

An 18-year-old man was taken to our hospital emergency room by a 120 ambulance after his motorcycle collided with a taxi on October 12, 2021. Digital radiography and whole-body computed tomography (CT) showed left tibia and fibula open fractures, rib fractures, patella fracture, scapula fracture, acetabular fracture, femoral neck fracture, intertrochanteric fracture, and posterior hip dislocation (Fig. 1). Immediately, we placed traction on the injured limb to prevent further displacement of the fracture and damage to the blood supply to the femoral head. Preoperative traction failed to correct the posterior hip dislocation, and the position of the femoral head remained unchanged. After 1 week, the patient general condition stabilized, we decided to perform surgery for the patient, and we decided to use the Kocher-Langenbeck approach in the lateral decubitus position (Fig. 2). Because this patient had an intertrochanteric fracture and ipsilateral femoral neck fracture, a proximal femoral anatomic plate and cannulated screws were used to fix the proximal femur. Accurate reduction of the femoral neck fracture was performed, and the fracture was compressed with 2 cannulated screws. Notably, we also reconstructed the blood supply of the femoral head with a quadratus femoris bone flap graft. The type of acetabular fracture was a fracture of the posterior wall of the acetabulum, and we performed anatomic reduction of the fracture and fixation with reconstructive plates and cannulated screws (Fig. 3).

**Figure 4.**
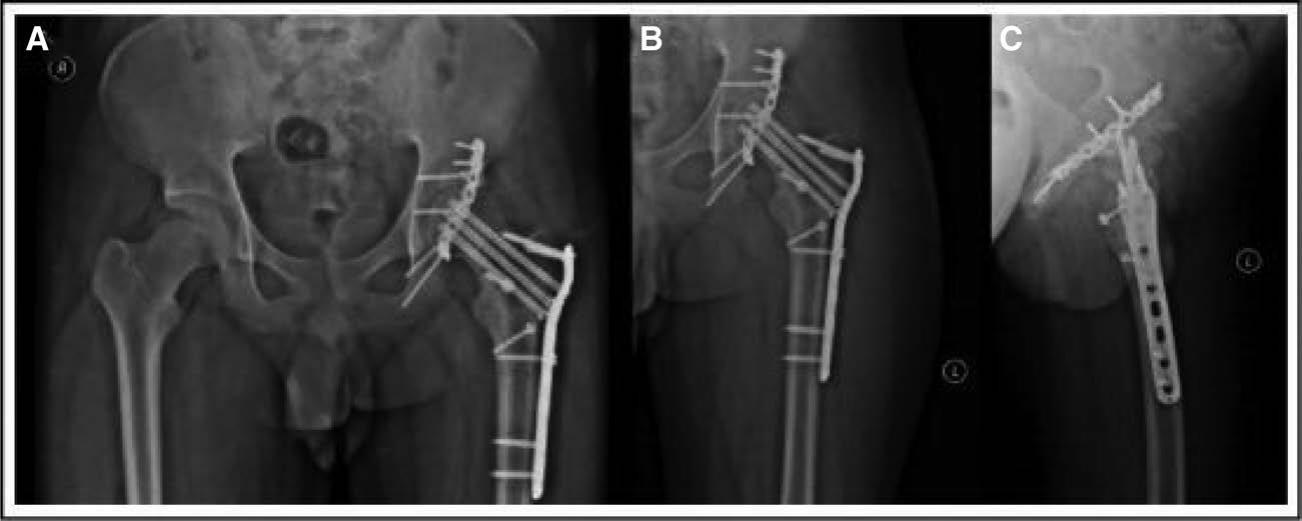
Three months after the operation. (A) The patient review showed that the internal fixation position was consistent with that after the operation, with no collapse and necrosis of the femoral head, no sclerosis of the acetabulum, and a small amount of callus formation, and the fracture line became more blurred than before. (B) Anteroposterior DR image of the left hip joint; (C) lateral DR image of the left hip joint. DR = digital radiography.

**Figure 5.**
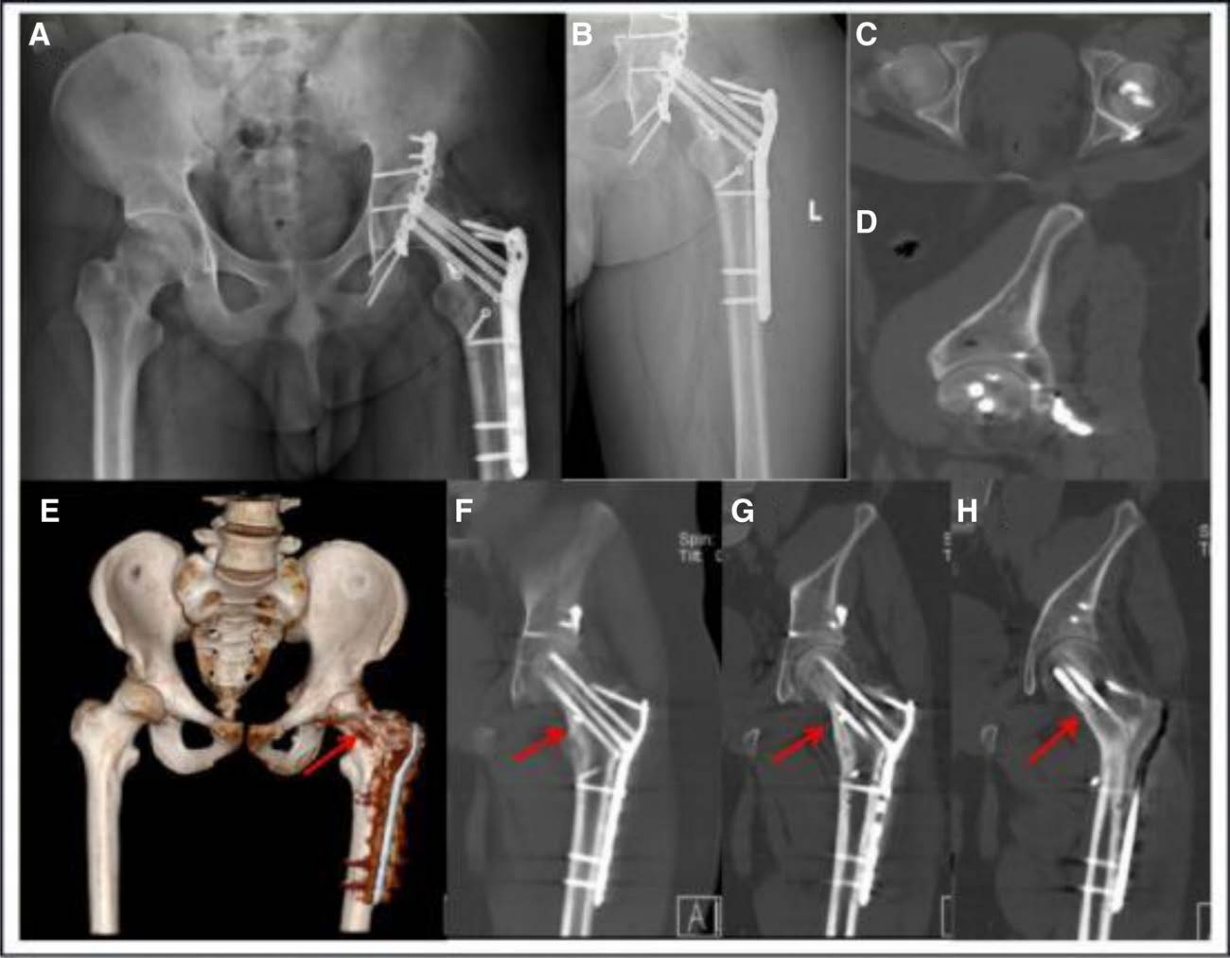
The patient review 6 months after surgery showed that the internal fixation position was consistent with that after surgery, with no collapse and necrosis of the femoral head, no sclerosis of the acetabulum, large callus formation, and a more blurred fracture line than before. (A) Anteroposteric DR image of the pelvis; (B) anteroposterior DR image of the left hip joint; (C) CT scan image of hip joint cross-section; (D) sagittal CT scan of the hip joint; (E) anteroposteric CT 3-dimensional reconstruction of the pelvis, the length of the femoral neck was basically the same on both sides, and there was more callus formation at the proximal end of the left femur; (F, G, H) Sagittal CT scan of the left hip joint, the fracture line of the proximal femur was blurred, the fracture of the femoral neck was well healed, and no collapse or necrosis of the femoral head was observed. DR = digital radiography.

**Figure 6.**
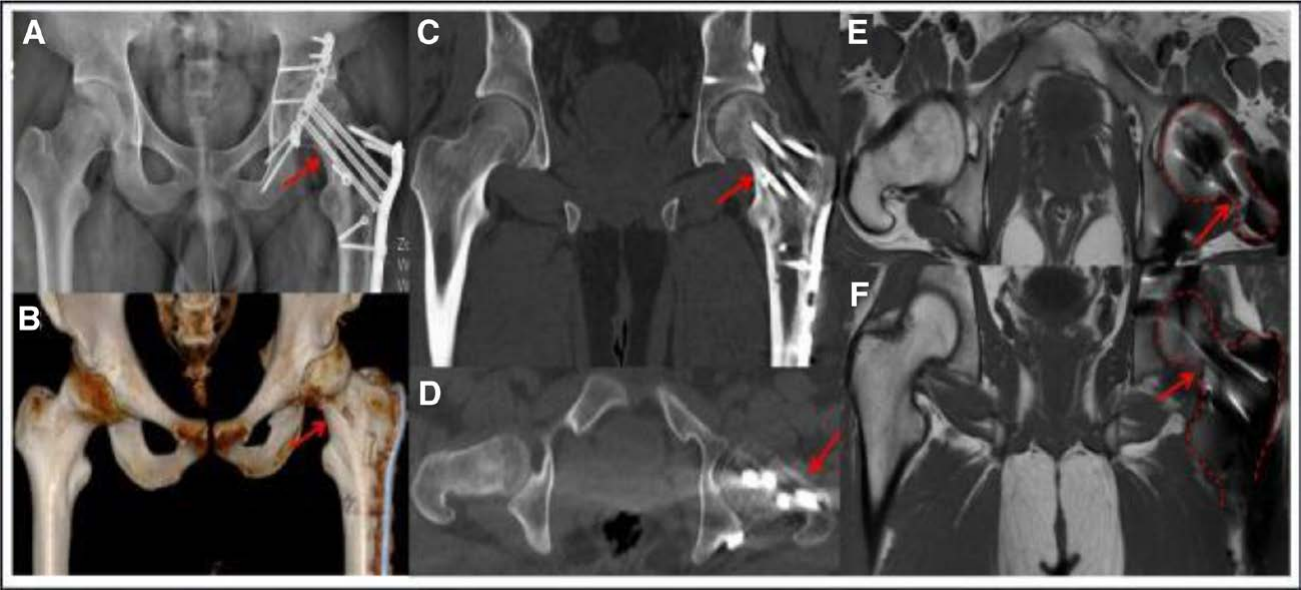
Twenty months after the operation, the patient internal fixation position was consistent with that after the operation. No collapse or necrosis of the femoral head was observed, no hardening of the acetabulum was observed, a large number of callus formed, and the fracture line disappeared. (A) Forward X-ray images of the pelvis; (B) CT 3-dimensional reconstruction of the pelvis; (C) coronal CT scan images of the hip joint; (D) CT scan images of hip joint transversal; (E) MRI images of the transverse hip joint, with red dotted lines representing the femoral head and femoral neck; (F) coronal MRI images and scanning images of the hip joint showed blurred fracture line of the proximal femur, good fracture healing of the femoral neck, and no collapse or necrosis of the femoral head.

Postoperatively, the patient remained in a position of mild flexion and abduction of the left hip joint during the 4-week bedridden period and then ended the bedridden period and entered an intensive 2-week rehabilitation program. Six weeks after surgery, he was able to walk on crutches without weight bearing on his left lower extremity. On discharge at 12 weeks postoperatively, the patient did not complain of significant left leg pain, and left hip joint function was satisfactory. He followed the physical therapy instructions that we suggested for home rehabilitation to strengthen muscles and keep joints moving. DR and CT examinations were performed 3 months (Fig. 4) and 6 months (Fig. 5) after surgery, and the findings indicated that the fracture was healing. At 20 months after surgery, DR, CT, and MRI of the patient hip indicated that the fracture had completely healed (Fig. 6).

**Figure 7.**
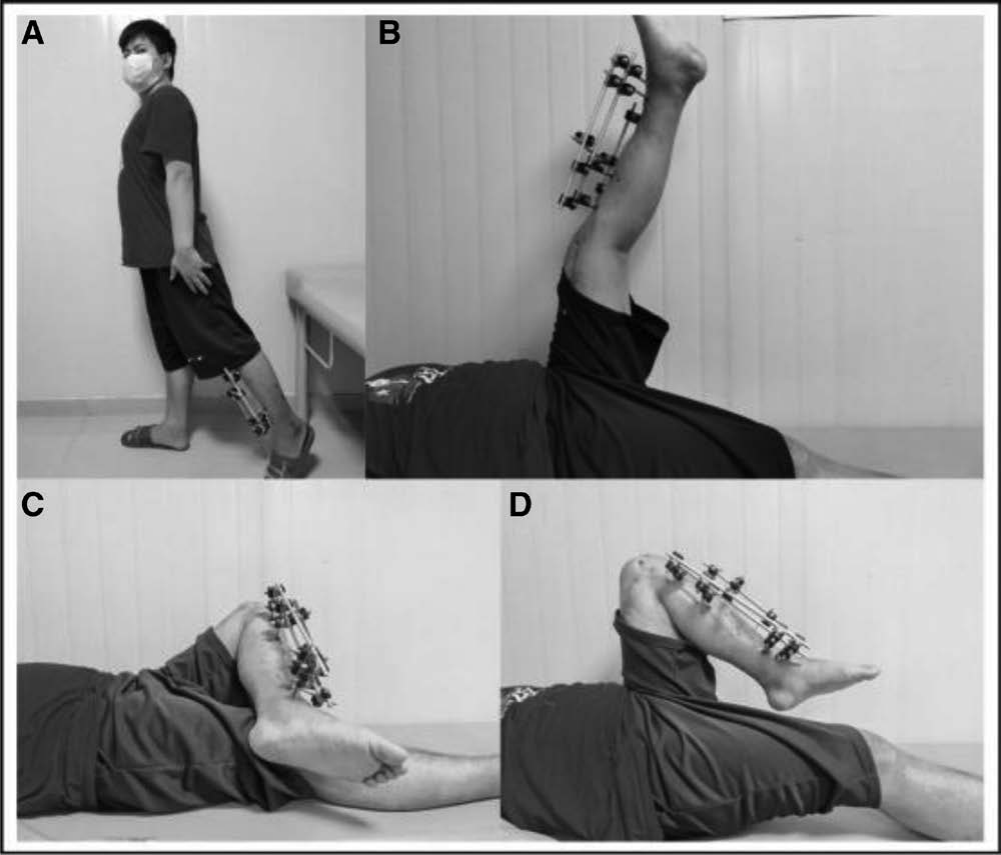
The hip function of the patient 6 months after the operation was as follows: (A) the range of hip motion was 0° to 110° in flexion, 0° to 10° in extension, 0° to 30° in abduction, 0° to 20° in adduction, and slight limitation in internal and external rotation. (B) Supine position with the straight knee joint and flexion hip joint; (C) supine position, flexion of the knee joint and abduction of the hip joint; (D) supine position, knee flexion, and maximum hip flexion.

**Figure 8.**
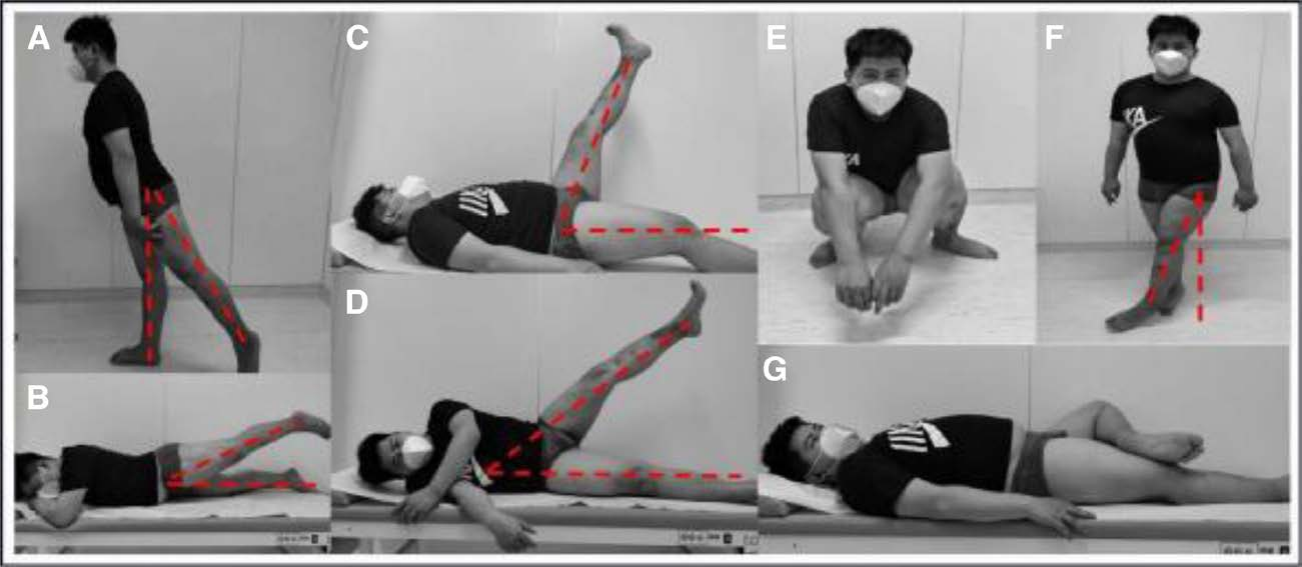
Hip function at 20 months after surgery. (A, B) Posterior extension of the hip joint in standing and prone positions; (C) hip straight leg elevation in the supine position; (D) hip abduction activity in the supine position; (E) the patient squatting activity; (F) adduction of the hip joint in standing position; (G) hip joint “4” test.

When we followed up with the patient at 6 months after surgery, the fracture union condition was evaluated by DR and CT examination and clinical symptoms. Clinical symptoms of pain with weight bearing, range of motion in the left hip joint and radiographic results were evaluated. His left hip joint range of motion was 0° to 110° in flexion, 0° to 10° in extension, 0° to 30° in abduction, 0° to 20° in adduction, and slightly limited in internal and external rotation, and his Harris Hip Score was 90 (Fig. 7). DR examination did not reveal traumatic arthritis. The proximal femur fractures and acetabulum fractures healed almost completely, with no sign of femoral head necrosis, despite mild limitation of hip motion. Six months after surgery, the patient was able to walk and carry out daily activities without any support. At 20 months postoperatively, the function and healing of the affected hip was comprehensively assessed by physical examination, DR, CT, and MRI. The Harris hip score was 94 points (Fig. 8), which included: normal appearance (4 points), no pain or discomfort (44 points), mild limping without any assistive tools (19 points), one-time maximal walking distance of more than 9 kilometers (8 points) The hip joint range of motion is 0° to 120° of flexion, 0° to 20° of extension, 0° to 45° of abduction, 0° to 20° of adduction, and 0° to 40° of internal and external rotation (5 points). We obtained informed consent from the patient and his guardian for publication of this case report.

## 3. Discussion

Ipsilateral hip dislocation with femoral neck fracture, intertrochanteric fracture, and acetabular fracture is a rare and serious injury that usually occurs after high-energy trauma. Traffic accidents and falls are common causes of such complex injuries. In our case, we learned from the accident report that he was hit by a car from the side while riding a motorcycle. In patients with multiple traumas, musculoskeletal injury is the most common lesion and almost always requires surgical treatment, and these patients often face challenges in functional prognosis and quality of life.^[11,12]^ We think that intertrochanteric fractures with ipsilateral posterior wall acetabular fractures and posterior dislocation of the hip are more common; however, in this case, concomitant femoral neck fractures are relatively rare because posterior wall acetabular fractures and hip dislocations will cushion the greater impact force in the femoral neck region, and when intertrochanteric fractures with an intact posterior wall of the ipsilateral acetabulum are more likely to lead to ipsilateral femoral neck fractures. This also poses a challenge for our treatment. At present, there is no clear classification method for the injury of such fractures combined with dislocation. With the number of high-energy injuries increasing, the incidence of such complex injuries will also be increasing, and some scholars recommend the Thompson- and Epstein-type injury classification as a grading method for this type of complex hip fracture with dislocation.^[13]^ It is difficult to accurately diagnose this fracture by X-ray alone, but 3-dimensional reconstruction CT scans can obtain a rapid and accurate diagnosis in the emergency department.

Both femoral neck fracture and hip dislocation can lead to AVN, and it is well known that reduction after dislocation should be performed as soon as possible to minimize the risk of femoral head necrosis. However, simultaneous fractures and dislocations due to this high-energy injury often fail to achieve successful correction of the dislocation, especially when the patient health is too unstable for immediate surgery. Therefore, for such patients, the optimal timing of surgery and emergency management methods must be planned according to the patient physiological status and other injuries. It has been reported that delayed surgical fixation also predisposes patients to osteonecrosis of the femoral head (ONFH).^[14,15]^ Kuhn et al and Barrett et al pointed out that a 4- to 5-day interval between injury and surgery did not have a large effect on postoperative fracture healing.^[8,9]^ In addition, most surgeons propose a staged treatment strategy when dealing with such unstable polytrauma patients.^[8]^ The surgical treatment of intertrochanteric fracture, femoral neck fracture, and dislocation of the hip joint includes fracture internal fixation or hip arthroplasty.^[16,17]^ However, it has been pointed out in the literature^[18]^ that one-stage hip arthroplasty is not recommended for young patients, even if the type of injury is complex. Although successful surgical treatment has been reported, there is still no standard surgical treatment for this rare complex fracture type^.[19]^ We believe that all the factors of each patient must be taken into account when deciding the choice of internal fixation. Through a literature review, 2 young patients were reported, both of whom were fixed with a dynamic hip screw.^[4,5]^ However, none of these patients received bone flaps. One of the patients, postoperatively, was kept on longitudinal traction through an upper tibial skeletal pin for 6 weeks, after which mobilization was initiated. At 3 years of regular follow-up, the patient was able to walk without pain and a limp, and radiography at 3 years showed good fracture healing without any sign of ONFH or heterotrophic ossification. In another patient, at 16 weeks, the femoral neck and the greater trochanter showed radiological union with good hip function. At the 2-year follow-up, early radiological signs of avascular necrosis appeared, but the patient continued to have a relatively painless hip with good function.

In our case, because the patient was young, we ultimately chose one-stage ipsilateral acetabular fracture internal fixation and femoral head vascular reconstruction surgery rather than hip arthroplasty. We utilized 2 cannulated lag screws and 1 cannulated lag screw through the PFLCP to fix the femoral neck fracture and the PFLCP to fix the intertrochanteric fracture (it can slide and compress the femoral neck and fix the intertrochanteric fracture at the same time). In contrast, the dynamic hip screw allows controlled dynamic sliding of the head element along the construct. However, the combined 3-part femur fracture is extremely unstable, and dynamic sliding compression may lead to ipsilateral femoral neck shortening or displacement. However, the patient treatment time was delayed due to the simultaneous open fracture of the ipsilateral tibia and fibula and systemic hemodynamic instability after injury. The open surgical approach is almost necessary for the treatment of such complex fractures and can effectively decompress the intracapsular hematoma, but this may also disrupt the blood supply to the femoral head, thereby increasing the risk of AVN.^[20]^ For simultaneous reduction and fixation of the posterior wall fracture of the acetabulum, the patient was operated on in lateral decubitus via a Kocher-Langenbeck approach. Additionally, we performed concomitant quadratus muscle bone flap transplantation during surgery to reconstruct the blood supply to the femoral head. Usually, the collapse of the femoral head and the appearance of traumatic osteoarthritis are manifestations of ONFH, but in our case, neither DR nor CT examination revealed the appearance of traumatic coxarthritis, collapse of the femoral head, or internal fixation loosening. Although the patient hip motion was somewhat limited, the follow-up results suggested that we cured his proximal femur fracture and other fractures. Six months after surgery, the patient was able to walk and perform daily activities without any support. This phenomenon differs from the previously reported higher incidence of ONFH. Therefore, we propose a strategy without staged treatment. The key to treatment is to reconstruct the blood supply of the femoral head with the inclusion of microscopic techniques while performing primary internal fixation of proximal femoral fracture, which may be useful for the treatment of patients with this complex unstable multitraumatic fracture.

## 4. Limitation

There are also some limitations to this study. Firstly, this is a case report and may not have taken into account that important basic information and potential risk factors can vary in some different populations of patients. Second, for this severe and complex injury, patients usually have additional injuries, but we did not discuss much in our analysis whether these additional injuries may have a greater impact on the healing of femoral neck fractures. Thirdly, our review of the literature identified hip dislocation as an independent risk factor for poor prognosis in this patient population.^[21]^ However, our case was not successfully reduced immediately after the injury; therefore, we cannot determine whether early reduction could help to reduce the incidence of avascular necrosis and further improve the patient prognosis.

## 5. Conclusion

For patients with complex ipsilateral femoral neck fracture, intertrochanteric fracture, acetabular fracture and hip dislocation, the key to surgical treatment is not only anatomical reduction and good fixation but also primary reconstruction of the blood supply to the femoral head, which may effectively reduce the incidence of femoral head necrosis and promote fracture healing.

## Author contributions

**Writing – original draft:** Dong Liu, Xiaojun Yu, Lin Chen.

**Writing – review & editing:** Zhiqiang Wang.
